# Mitigating Effects of *Liriope platyphylla* on Nicotine-Induced Behavioral Sensitization and Quality Control of Compounds

**DOI:** 10.3390/brainsci10090654

**Published:** 2020-09-21

**Authors:** Dahye Yoon, In Soo Ryu, Woo Cheol Shin, Minhan Ka, Hyoung-Geun Kim, Eun Young Jang, Oc-Hee Kim, Young-Seob Lee, Joung-Wook Seo, Dae Young Lee

**Affiliations:** 1 Department of Herbal Crop Research, National Institute of Horticultural and Herbal Science, RDA, Eumseong 27709, Korea; dahyeyoon@korea.kr (D.Y.); shinwoocheol@korea.kr (W.C.S.); youngseoblee@korea.kr (Y.-S.L.); 2 Pharmacology and Drug Abuse Research Group, Korea Institute of Toxicology, Daejeon 34114, Korea; insoo.ryu@kitox.re.kr (I.S.R.); minhan.ka@kitox.re.kr (M.K.); eunyoung.jang@kitox.re.kr (E.Y.J.); ochee.kim@kitox.re.kr (O.-H.K.); 3 Department of Oriental Medicinal Biotechnology and Graduate School of Biotechnology, Kyung Hee University, Yongin 17104, Korea; zwang05@khu.ac.kr

**Keywords:** *Liriope platyphylla*, saponin, nicotine, dopamine transporter, behavioral sensitization, quality control

## Abstract

In this study we investigated the mitigating effects of *Liriope platyphylla* Wang et Tang extract on behavioral sensitization and the quantification of its major compounds. The extract of *L. platyphylla* reduces the expression of tyrosine hydroxylase (TH) protein, which is increased by nicotine, back to normal levels, and increases the expression of dopamine transporter (DAT) protein, which is reduced by nicotine, back to normal levels in PC12 cells. In this study, rats received nicotine (0.4 mg/kg, subcutaneously) only for seven days and then received extract of *L. platyphylla* (200 or 400 mg/kg, oral) 1 h prior to nicotine administration for an additional seven days. The extract of *L. platyphylla* reduced locomotor activity compared to the nicotine control group in rats. The extract of *L. platyphylla* significantly attenuated the repeated nicotine-induced DAT protein expression in the nucleus accumbens (NAc), but there was no effect on increased TH protein expression in the dorsal striatum. These findings suggest that *L. platyphylla* extract has a mitigating effect on nicotine-induced behavioral sensitization by modulating DAT protein expression in the NAc. For quality control of *L. plathyphylla*, spicatoside A and D, which are saponin compounds, were quantified in the *L. platyphylla* extract. The amounts of spicatoside A and D in *L. platyphylla* extract obtained from ultra-high-performance liquid chromatography with tandem mass spectrometry were 0.148 and 0.272 mg/g, respectively. The identification of these compounds in *L. platyphylla*, which can be used for quality control, provides important information for the development of drugs to treat nicotine dependence.

## 1. Introduction

Cigarette smoking is one of the major global public health problems. Nicotine, a major active alkaloid in tobacco products, acts as a psychoactive compound in the brain and causes alterations in the dopaminergic neurotransmission of the mesolimbic reward system [1,2], leading to behavioral sensitization [2,3]. The dorsal striatum (dSTR) and nucleus accumbens (NAc) are major integration regions of the basal ganglia circuitry which receive a large variety of projections including dopaminergic inputs from the substantia nigra and ventral tegmental area, respectively [4]. The dSTR and NAc play important roles in nicotine-induced behavioral sensitization [4,5]. Tyrosine hydroxylase (TH) and dopamine transporter (DAT) are well-known modulators of dopamine concentrations in the reward system [6,7]. TH is a rate-limiting enzyme that regulates the synthesis of dopamine in the dopaminergic neurons, and DAT regulates synaptic dopamine concentration by the reuptake of dopamine into presynaptic terminals [6,7]. Previous investigations demonstrated that total ginseng saponin attenuates nicotine-induced alternations in dopaminergic neurotransmission in the reward system of the brain and mitigates nicotine-induced behavioral changes such as behavioral sensitization [2,5,8]. Behavioral sensitization—the hypersensitivity of motivated behaviors after repeated exposure to nicotine—is closely associated with alterations to dopaminergic neurotransmission in the reward system [2,9,10]. Controlling dopaminergic neurotransmission is considered important to attenuate nicotine-induced behavioral sensitization. However, the effects of *L. platyphylla* on the dopaminergic system and nicotine-induced behavioral changes have not yet been fully characterized.

*L. platyphylla* is a perennial evergreen herb distributed across Korea, China, India, and Japan [11]. The medicinal effects of *L. platyphylla* on asthma, lung inflammation, brain associated diseases, and constipation were recorded in the Korean medical classic *Donguibogam*, which was nominated as Memory of the World by United Nations Educational, Scientific and Cultural Organization (UNESCO) in 2009, and these effects have been confirmed using modern scientific studies [12,13,14]. In addition to these effects, the homoisoflavones contained in *L. platyphylla* affect diabetes by enhancing insulin action [15], and the effect of obesity on *Gyeonshingangjeehwan* (GGEx), which includes *L. platyphylla*, was also confirmed [16]. *L. platyphylla* was reported to exhibit neuroprotective [12] and hepatoprotective effects [17]. *Liriope* spp. contain many saponin compounds such as spicatoside A, B, C, and D; and ophiopogonin A, B, C, and D. Spicatoside A, the major component of *L. platyphylla*, was reported to have anti-inflammatory, anti-asthma, anti-osteoclastogenesis, neurite outgrowth, memory consolidation, and anti-cancer effects [18]. While its chemical structure differs from ginseng saponin [8], which has been shown to have a blocking effect against nicotine-induced behavioral sensitization, here we examine whether the same effect exists in *L. platyphylla*, which contains saponin compounds.

It is difficult to distinguish between *L. platyphylla* and *Ophiopogon japonicus* on the basis of appearance alone, which presents a problem in terms of quality evaluation and control. Shin [19] reported that the composition and contents of the saponins in the two species differed, with *L. platyphylla* having higher spicatoside A content. Another study examined the content of spicatoside A from the roots of *L. platyphylla* [20], which also contained a high amount of spicatoside D. Based on these findings, spicatoside A and D can be considered important with regard to quality evaluation and control of *L. platyphylla.*

In this study, the mitigating effects of *L. platyphylla* extracts, which contain saponin compounds, on the dopaminergic system and repeated nicotine-induced behavioral sensitization, were evaluated using in vitro and in vivo approaches. The saponin compounds, spicatoside A and D, in *L. platyphylla* were quantitatively analyzed.

## 2. Materials and Methods 

### 2.1. Preparation of L. platyphylla Extract

Roots of *L. platyphylla* were purchased from the Korea Medicine Herbal Association (Seoul, Korea) and the materials were cultivated in Cheongyang province by authentication. For the in vitro and in vivo studies, *L. platyphylla* roots were coarsely ground and extracted twice by reflux extraction with H_2_O at 80 °C for 4 h. The extract was dried after filtering using filter paper. For quantitative analyses of spicatoside A and D, *L. platyphylla* roots were ground and extracted ultrasonically with methanol at 40 °C for 1 h. The extract was filtered through a 0.22 μm polytetrafluoethylene (PTFE) membrane syringe filter and lyophilized. Then, 400 mg of the extract was re-suspended in 10 mL H_2_O and extracted twice with 10 mL *n*-butanol (*n*-BuOH). The *n*-BuOH layer was concentrated under reduced pressure. For quantifying small amounts of compounds, *L. platyphylla* roots were homogenized using a ball mill (MM400, Retsch, Haan, Germany). Fine powder of *L. platyphylla* (100 mg) was suspended in 1 mL of methanol and ultrasonically extracted for 1 h at 40 °C. The extract was centrifuged at 15,000 rpm for 5 min at 4 °C, and the supernatant was filtered through a 0.22 μm PTFE membrane syringe filter.

### 2.2. Preparation of Sample and Standard Solutions

Spicatoside A and D isolated from the roots of *L. platyphylla* grown in Cheongyang, South Korea, were identified using medium-pressure liquid chromatography (MPLC) and NMR techniques (results published elsewhere). Two accurately weighed standard compounds were dissolved in methanol to prepare stock solutions containing 1.00 mg/mL. The stock solutions were diluted with methanol to obtain calibration solutions in the range of 0.019–5 μg/mL. Finally, diluted stock solutions were filtered through 0.22 μm PTFE membrane syringe filter and kept at 4 °C. Nicotine tartrate was purchased from Thermo Fisher Scientific (Waltham, MA, USA). Nicotine was dissolved in phosphate-buffered saline (PBS, pH 7.4) for the in vitro study or 0.9% physiological saline for the in vivo study. The extract of *L. platyphylla* was dissolved in distilled water for the in vitro and in vivo studies. All solutions were freshly prepared just before each experiment.

### 2.3. Analysis of L. platypylla Extract Using UPLC-QTOF/MS

Ultra-high-performance liquid chromatography (UPLC) was performed on a Waters ACQUITY H-Class (Waters Corp., Milford, MA, USA) using a HALO C18 column (150 × 2.1 mm, 2.7 μm; Advanced Material Technology, Wilmington, DE, USA) for chromatographic separation. The column temperature was maintained at 40 °C. The mobile phase composed of water containing 0.1% formic acid (*v*/*v*, solvent A) and acetonitrile containing 0.1% formic acid (*v*/*v*, solvent B) was pumped at a flow rate of 0.3 mL/min and the injection volume was 2 μL. The gradient elution program was set as follows: 0–10 min, B 20–32%; 10–11 min, B 32–45%; 11–25 min, B 45–50%; 25–27 min, B 50–65%; 27–28 min, B 65–20%; 38–30 min, 20%. Mass analysis was performed using a Xevo G2-S quadrupole time-of-flight (QTOF) mass spectrometer (Waters Corp.) in negative ion mode (ESI–). Accurate mass was measured using an automatic calibrated delivery system with internal reference (leucine-enkephalin, *m/z* 554.2615 (ESI–)). The operational parameters were set as shown in Table 1.

### 2.4. Quantitative Analyses of Standard Compounds Using UPLC-MS/MS

For the quantitative analyses of spicatoside A and D in the 100% methanol extract of *L. platypylla*, UPLC with tandem mass spectrometry (MS/MS) was conducted. UPLC was performed on a Waters ACQUITY UPLC instrument (Waters Corp.) with a HALO RP-Amide column (150 mm × 2.1 mm, 2.7 μm; Advanced Material Technology). The mobile phase was water (solvent A) and acetonitrile (solvent B). The elution gradient was as follows: 0–6 min, B 15–40%; 6–20 min, B 40–60%; 20–22 min, B 60–65%; 22–23 min, B 65–100%; 23–25 min, B 100–15%, and 25–26 min, B 15–15%. The injection volume was 2.0 μL, the temperature of the column oven was 40 °C, and the flow rate was 0.3 mL/min for each run. Mass spectrometric detection was conducted on a 3200 QTRAP mass spectrometer (AB SCIEX, Framingham, MA, USA) using ESI– with multiple reaction monitoring (MRM) detection. Precursor ions of separated compounds were selected, collision energies were applied to selected ions, and product ions were used for quantification. The optimal operating parameters were set as shown in Table 2.

### 2.5. Cell Culture

Since the PC12 cell line expresses TH and DAT proteins well [21,22], we used the PC12 cell line in this study. The PC12 cell line was purchased from American Type Culture Collection (University Boulevard Manassas, VA, USA). PC12 cells were cultured and maintained in Dulbecco’s Modified Eagle’s Medium (DMEM) supplemented with heat-inactivated 5% horse serum, 10% fetal bovine serum, and 1% streptomycin/penicillin in a humidified atmosphere at 37 °C and 5% CO_2_, as previously described [21].

### 2.6. Cell Viability Test

Cell viability was determined by the conventional 3-(4,5-dimethylthiazol-2-yl)-5-(3-carboxymethoxyphenyl)-2-(4-sulfophenyl)-2H-tetrazolium (MTS) assay. PC12 cells were plated in 96-well plates at a density of 1 × 10^4^ cells/well in DMEM and cultured for additional 24 h. The cells were treated with various concentration of *L. platyphylla* extracts (1, 10, 100, and 1000 μg/mL) or nicotine solution (1, 10, 100, and 1000 μM/mL) for additional 24 h. After incubation, cells were treated with the MTS solution (3-(4,5-dimethylthiazol-2-yl)-5-(3-carboxymethoxy phenyl)-2-(4-sulfophenyl)-2H-tetrazolium, inner salt) for 1 h at 37 °C. The reaction was stopped by adding stop solution (10% sodium dodecyl sulfate, SDS), and the absorbance was measured at 490 nm by using a Gloamax-Bultimicro plate multimode Reader (Promega, Madison, WI, USA). 

### 2.7. Western Immunoblotting

Western immunoblotting was performed as previously described [23]. For western immunoblotting, PC12 cells were plated in 6-well plates at a density of 1 × 10^4^ cells/well in the culture medium for 24 h. The treated doses of *L. platyphylla* extract and nicotine following in vitro study were determined from the results of a cell viability test. Cells were co-treated with *L. platyphylla* extracts (10 μg/mL) and a nicotine solution (1000 μM/mL) for an additional 24 h. After incubation, cells were collected in 0.1 mL of a mixture of radioimmunoprecipitation assay (RIPA) buffer and protease and phosphatase inhibitor cocktails (Thermo Fisher Scientific), after which they were incubated at 4 °C for 30 min. After incubation, lysates were centrifuged at 13,000 rpm for 30 min at 4 °C. The pellet, which primarily contained nuclei and large debris, was discarded and the supernatant was obtained. The concentration of the solubilized proteins in the supernatant fraction was determined based on the bicinchoninic acid (BCA) assay using a BCA assay kit (Thermo Fisher Scientific). The proteins in the supernatant fraction were resolved using 10% bisacrylamide gel electrophoresis and then the separated proteins were transferred to a polyvinylidene fluoride membrane (PVDF; Bio-Rad, Hercules, CA, USA). The protein-transferred PVDF membrane was treated with blocking buffer containing 5% bovine serum albumin in a mixture of Tris-buffered saline and 0.1% Tween-20 (TBST), and washed three times for 10 min each with TBST. After washing, the membrane was probed with a rabbit primary antiserum for tyrosine hydroxylase (TH, 1:1000, Cell Signaling Technology, Danvers, MA, USA) and dopamine transporter (DAT, 1:1000; abcam, Cambridge, MA, USA) for 18 h at 4 °C on a shaker. Then, the membrane was re-washed three times, and incubated with goat anti-rabbit immunoglobulin G horse radish peroxidase-labeled secondary antiserum (Thermo Fisher Scientific) for 1 h at room temperature. Immunoreactive protein bands were detected by enhanced chemiluminescence reagents (Thermo Fisher Scientific) using Image Lab software (version 5.2.1, Bio-Rad). After stripping, the same membranes were re-probed with a mouse primary antiserum for β-actin (1:2000; #A5316, Sigma-Aldrich, St. Louis, MO, USA) for blot normalization. Immunoreactive protein bands on the membrane were semi-quantified using ImageJ software (version 1.52a, National Institutes of Health, Bethesda, MD, USA). 

### 2.8. Animals

Adult male Sprague–Dawley rats weighing between 200 and 230 g (6 weeks old) were purchased from Orient Bio. Inc. (Seongnam, South Korea). Rats were separated into pairs and acclimated to their home cages for a minimum of 5 days. Food and water were provided ad libitum. Animals were housed in a temperature (21–23 °C) and humidity (45–55%) controlled vivarium under a 12/12 h light/dark cycle (light on at 8:00 a.m.). Experimental treatments were applied in a quiet room to minimize environmental stress. All animal procedures were approved by the Institutional Animal Care and Use Committee of Korea Institute of Toxicology (Approval No. 1811-0443) and conducted in accordance with the provisions of the Guide for the Care and Use of Laboratory Animals issued by the U.S. National Institute of Health. 

### 2.9. Administration of Nicotine and L. platyphylla Solution

The route and dose of nicotine administration were determined by our previous studies [5]. All rats were subcutaneously (s.c.) administered saline (1.0 mL/kg) or nicotine (0.4 mg/kg/day, 1.0 mL/kg) for 14 consecutive days. From the 8th day, rats were pretreated with vehicle or *L. platyphylla* solution (0, 200, or 400 mg/kg/day, 1 mL/kg/day) orally (p.o.) 1 h prior to each saline or nicotine administration. Rats were randomly divided into six groups as follows: (1) repeated saline (7 days) + pretreatment of vehicle prior to saline (7 days) group (*n* = 7); (2) repeated saline (7 days) + pretreatment of *L. platyphylla* extract (200 mg/kg) prior to saline (7 days) group (*n* = 7); (3) repeated saline (7 days) + pretreatment of *L. platyphylla* extract (400 mg/kg) prior to saline (7 days) group (*n* = 7); (4) repeated nicotine (7 days) + pretreatment of vehicle prior to nicotine (7 days) group (*n* = 7); (5) repeated nicotine (7 days) + pretreatment of *L. platyphylla* extract (200 mg/kg) prior to nicotine (7 days) group (*n* = 7); and (6) repeated nicotine (7 days) + pretreatment of *L. platyphylla* extract (400 mg/kg) prior to nicotine (7 days) group (*n* = 7).

### 2.10. Locomotor Activity

The behavioral assessment for locomotor activity test was performed as described previously [5]. Rats were acclimated to a square seamless open-field arena (43 × 43 × 30 cm, #ENV-515S, Med Associates, Georgia, VT, USA) in an illuminated and sound-attenuated cubicle (#SAC-283422-NIR, Med Associates) for at least 3 days (for 30 min/day) to avoid the influence of environmental factors prior to experiments. Locomotor activity was recorded using a computer-based monochrome/near infrared video camera (#VID-CAM-MONO-1, Med Associates). On the test day, to determine the mitigating effects of *L. platyphylla* on nicotine-induced locomotor activity, rats were pretreated with vehicle or *L. platyphylla* extract solution 1 h before measuring locomotor activity. After pretreatment, rats were placed in the open-field arena and basal activity was measured for 30 min. Rats were given the final administration of saline or nicotine after recording basal activity. Locomotor activity was recorded for an additional 1 h. Changes in locomotor activity were quantified using a computer-based video tracking system (Ethovision XT14, Noldus, Wageningen, The Netherlands) and values are expressed as total distance traveled (centimeter) for 1 h.

### 2.11. Brain Tissue Collection

After the measurement of locomotor activity for 1 h following the final saline or nicotine administration, rats were deeply anesthetized with 2–4% isoflurane, decapitated, and brains were rapidly removed. Tissue sections were serially cut using a stainless-steel coronal brain matrix (Roboz Surgical Instrument Co., Inc., Gaithersburg, MD, USA) at 4 °C. The dSTR and NAc were collected bilaterally for western immunoblotting analysis in vivo. Tissues were transferred to RIPA buffer with protease and phosphatase inhibitor cocktails, homogenized, and were then incubated at 4 °C for 30 min. The detailed western blotting procedures were the same as described above in the western immunoblotting section.

### 2.12. Statistics

Data are represented as mean ± standard error of the mean (SEM) of cell viability, immunoreactivity of protein expression in western blotting and travelled distance in the behavioral sensitization test. Tukey’s post hoc test was used for all one-way analysis of variance (ANOVA) with repeated measures, and Bonferroni’s post hoc test was used for all repeated measures two-way ANOVA. Statistical analysis was conducted using GraphPad Prism 8 (GraphPad Software, La Jolla, CA, USA). Statistical significance was accepted for *p*-values < 0.05. Each experiment of quantitative analyses was performed in triplicate. The data are reported as the mean ± standard deviation (SD) and were analyzed by SPSS (version 19.0, IBM Inc., Armonk, NY, USA).

## 3. Results

### 3.1. Analyses of Standard Compounds in L. platyphylla Extract by a UPLC-MS System

The extract of *L. platyphylla* was analyzed using optimized UPLC-QTOF/MS. Spicatosides A and D were separated by the UPLC system. 

**Figure 1:**
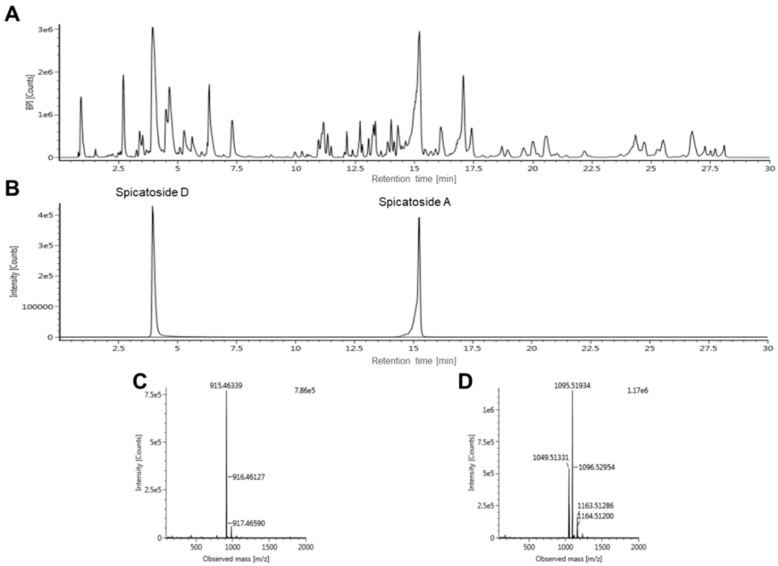
Ultra-high-performance liquid chromatography coupled with quadrupole time-of-flight mass spectrometry (UPLC-QTOF/MS) in negative-ion mode analysis. (**A**) Base peak intensity (BPI) chromatogram of *Liriope platyphylla* extract (*n*-butanol fraction), (**B**) overlay chromatogram of two standard compounds, (**C**) mass spectrum of spicatoside A, and (**D**) mass spectrum of spicatoside D.

Fractionation was performed because we found low intensities of overall compounds in the extract. After fractionation, two standards were confirmed in the UPLC chromatogram of the *n*-butanol fraction. A typical base peak intensity (BPI) chromatogram and the mass spectra of the identified standard compounds are shown in Figure 1. 

The intensities of spicatoside A and D were low in the *n*-butanol fraction for quantification using UPLC-QTOF/MS. Therefore, QTRAP^®^ MS/MS was used for quantifying small amounts of compounds. With the optimized instrument, the retention time of spicatoside A and D were 9.53 and 3.97 min, respectively. The precursor ions of spicatoside A and D for quantification using MRM mode were selected at *m/z* 869.385 and 1049.423, respectively (Table 3). After formation of optimum energies for fragmentation, the product ions with the best sensitivity were selected and quantified. The product ion of spicatoside A was *m/z* 737.4 and that of spicatoside D was *m/z* 917.2. Linear calibration curves were constructed using five different concentrations of two compounds. The values of the calibration plots are shown in Table 4. Spicatoside A and D showed excellent correlation coefficients.

### 3.2. Effects of L. platyphylla Extract and Nicotine on PC12 Cell Viability

**Figure 2:**
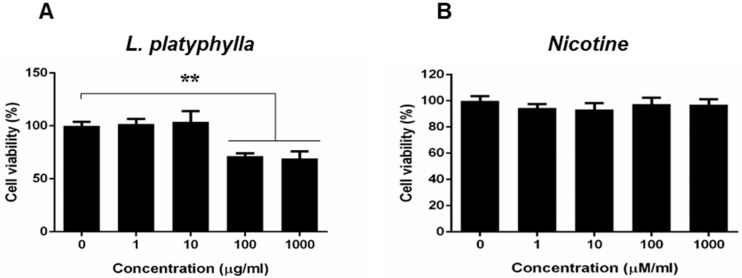
Dose-dependent cell viability of PC12 cells treated with *L. platyphylla* extract and nicotine. cytotoxicity of (**A**) *L. platyphylla* and (**B**) nicotine. One-way ANOVA followed by Tukey’s post hoc test was used for statistical analysis. ** *p* < 0.01 versus vehicle control. *N* = 3, independent cultures.

We performed a cell viability test to determine whether *L. platyphylla* extract and nicotine induce cytotoxicity in PC12 cells. The results showed that treatment with 100 and 1000 μg/mL of *L. platyphylla* extract, but not 1 and 10 μg/mL, significantly reduced cell viability in PC12 cells compared with the vehicle control group (*p* < 0.05; Figure 2A). However, various concentrations of nicotine treatment (1, 10, 100 and 1000 μM/mL) did not cause cytotoxicity in PC12 cells (Figure 2B). Based on these results, we used doses of *L. platyphylla* extract and nicotine as 10 μg/mL and 1000 μM/mL, respectively, in the following in vitro experiments.

### 3.3. Co-Treatment of L. platyphylla Extract with Nicotine Significantly Mitigated Nicotine-Induced Alterations in TH and DAT Protein Expression in PC12 Cells

**Figure 3:**
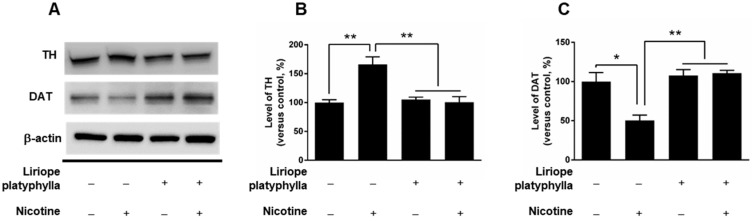
Effects of *L. platyphylla* on the nicotine-induced alterations in the expressed protein levels of tyrosine hydroxylase (TH) and dopamine transporter (DAT) in PC12 cells. (**A**) Representative blots for expression of TH and DAT proteins in PC12 cells. Quantification of expressed protein levels in (**B**) TH and (**C**) DAT 24 h after co-treatment of *L. platyphylla* and nicotine. One-way ANOVA followed by Tukey’s post hoc test was used for statistical analysis. * *p* < 0.05 and ** *p* < 0.01 versus vehicle + nicotine group. *N* = 3, independent cultures.

Since treatment with psychostimulants such as nicotine alters the expression level of TH and DAT in cell culture [24,25], we performed western immunoblotting to examine whether *L. platyphylla* extract attenuates the nicotine-induced alterations in the expression of TH and DAT protein in PC12 cells. The co-treatment of *L. platyphylla* extract with nicotine significantly mitigated the nicotine-induced increase in TH protein expression in PC12 cells compared to the vehicle control group (*p* < 0.05, Figure 3A,B). Similarly, the co-treatment of *L. platyphylla* extract with nicotine significantly rescued the nicotine-induced decrease in DAT protein expression in PC12 cells compared to the vehicle control group (*p* < 0.05; Figure 3A,C). However, treatment with *L. platyphylla* extract alone did not alter the levels of TH and DAT protein expression in PC12 cells (Figure 3A–C).

### 3.4. Pretreatment with L. platyphylla Extract Significantly Atteunuated Repeated Nicotine-Induced Increase in Locomotor Activity in Rats

**Figure 4:**
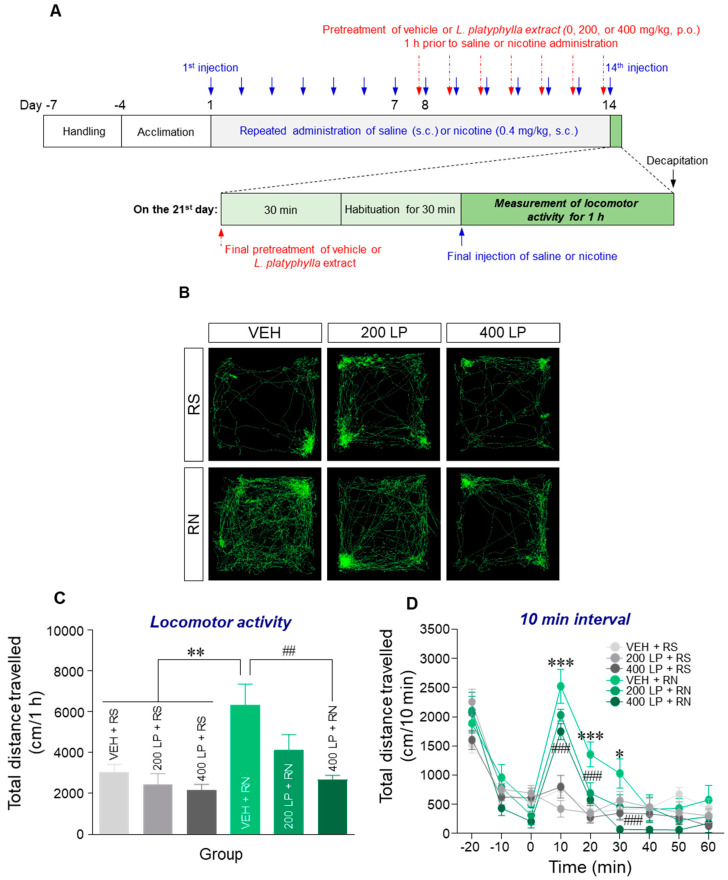
Effect of *L. platyphylla* extract on repeated nicotine-induced behavioral sensitization in rats. (**A**) Experimental timeline for behavioral assessment. Blue arrows represent subcutaneous injection of saline or nicotine. Red dotted arrows represent oral pretreatment of vehicle or *L. platyphylla* extract prior to each saline or nicotine injection. (**B**) Representative locomotion tracking patterns for 60 min after the final saline or nicotine administration. (**C**,**D**) Changes in locomotor activity are expressed as total distance travelled following the final administration of saline or nicotine after pretreatment of vehicle or *L. platyphylla* extract. One-way or two-way ANOVA, followed by Tukey’s post hoc test or Bonferroni’s post hoc test, respectively, were used for statistical analysis. * *p* < 0.05, ** *p* < 0.01, and *** *p* < 0.001 versus VEH + RS group. ^##^ *p* < 0.01 and ^###^ *p* < 0.001 versus VEH + RN group. RS, repeated saline; RN, repeated nicotine; VEH, vehicle; 200 LP, 200 mg/kg *L. platyphylla* extract; 400 LP, 400 mg/kg *L. platyphylla* extract.

Since repeated nicotine exposure induces behavioral sensitization [2,5], we measured locomotor activity to examine whether *L. platyphylla* extract attenuated the nicotine-induced behavioral sensitization in rats. The timeline for measuring locomotor activity following repeated saline or nicotine administration after the pretreatment with *L. platyphylla* extract is shown in Figure 4A. The results showed that repeated nicotine administration significantly increased locomotor activity (F(5, 36) = 6.50, *p* < 0.001; Figure 4B,C) compared with the repeated saline control group. 

The pretreatment with 400 mg/kg *L. platyphylla* extract, but not 200 mg/kg, prior to repeated nicotine administration, significantly decreased locomotor activity compared with the results from repeated nicotine administration followed by vehicle pretreatment (Figure 4B,C). In the analysis of post-administration in 10 min intervals, the pretreatment with 400 mg/kg *L. platyphylla* extract significantly decreased the repeated nicotine-induced increase in locomotor activity at 10, 20, and 30 min after the final nicotine administration (Time: F(8, 48) = 53.67, *p* < 0.001; Treatment: F(2, 12) = 5.01, *p* < 0.05; Time × Treatment: F(16, 96) = 1.76, *p* < 0.05) (10 min: F(2, 18) = 4.07, *p* < 0.05; 20 min: F(2, 18) = 6.11, *p* < 0.01; 30 min: F(2, 18) = 6.54, *p* < 0.01; Figure 4D). However, we found no significant differences in locomotor activity between vehicle and *L. platyphylla* extract pretreatment followed by repeated saline administration (Time: F(8, 48) = 33.28, *p* < 0.001; Treatment: F(2, 12) = 1.22, *p* = 0.33; Time × Treatment: F(16, 96) = 1.62, *p* = 0.07) (Figure 4C,D). The real values of changes in locomotor activity over 1 h after the final administration of saline or nicotine are listed in Table 5.

### 3.5. Pretreatment with L. platyphylla Extract Significantly Rescued Repeated Nicotine-Induced Decrease in the Level of DAT Protein Expression in the NAc of Rats

**Figure 5:**
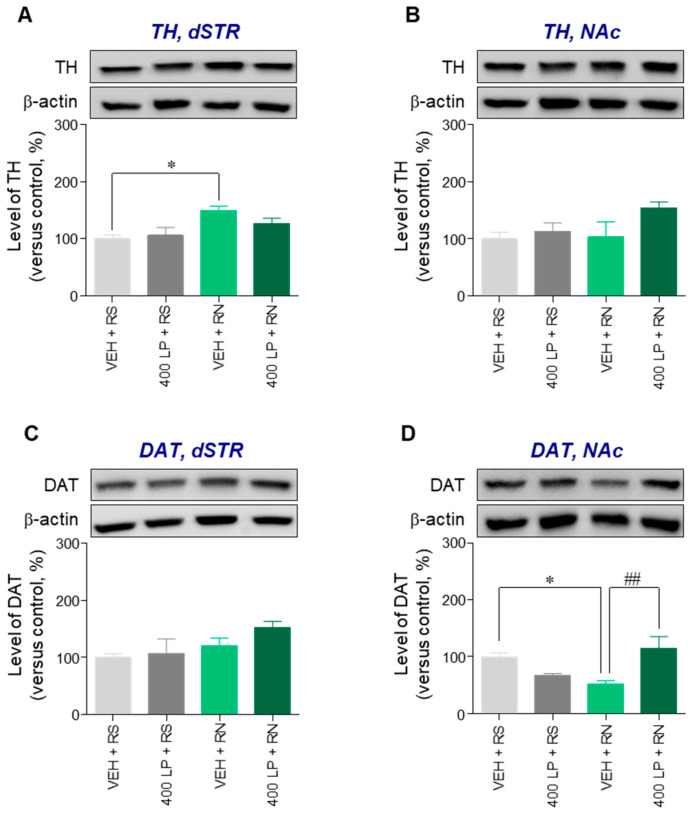
Effect of *L. platyphylla* extract on repeated nicotine-induced alternations in TH and DAT protein expression in the dorsal striatum (dSTR) and nucleus accumbens (NAc) of rats. (**A**–**D**) Representative blots and quantification of expressed protein levels in TH and DAT of the dSTR and NAc following the final administration of saline or nicotine after the pretreatment of vehicle or 400 mg/kg *L. platyphylla*. One-way ANOVA followed by Tukey’s post hoc test was used for statistical analysis. * *p* < 0.05 versus VEH + RS group. ^##^ *p* < 0.01 versus VEH + RN group. RS, repeated saline; RN, repeated nicotine; VEH, vehicle; 400 LP, 400 mg/kg *L. platyphylla* extract.

Since pretreatment of 400 mg/kg *L. platyphylla* extract significantly attenuated repeated nicotine-induced behavioral sensitization, we performed western immunoblotting to investigate whether *L. platyphylla* extract mitigates the repeated nicotine-induced alterations in TH and DAT expression in the dSTR and NAc. Repeated nicotine administration significantly increased the level of TH protein expression in the dSTR (F(3, 12) = 3.74, *p* < 0.05), but not NAc, compared to the repeated saline group (Figure 5A,B); however, there was no significant difference in the repeated nicotine-induced increase in TH protein expression of the dSTR between pretreatment of vehicle and *L. platyphylla* extract group (Figure 5A). There was no difference in DAT expression in the dSTR among groups, but the level of DAT expression in the repeated nicotine-treated group was significantly decreased compared with the repeated saline-treated group (*p* < 0.05) (Figure 5C,D). 

Additionally, the pretreatment of *L. platyphylla* extract significantly rescued the repeated nicotine-induced decrease in DAT protein expression in the NAc compared to the repeated nicotine group (F(3, 12) = 6.60, *p* < 0.01) (Figure 5D).

## 4. Discussion

We evaluated the mitigating effects of *L. platyphylla* extract on: (1) nicotine-induced alterations in TH and DAT protein expression in vitro and in vivo, and (2) repeated nicotine-induced behavioral sensitization. Tyrosine hydroxylase (TH) and dopamine transporter (DAT) are well-known modulators of dopamine levels in the reward system of the brain. TH is involved in the synthesis of dopamine and DAT controls dopamine levels by reuptake into presynaptic terminals [6,7]. A previous study demonstrated that treatment of ginseng containing saponins significantly rescued the nicotine-induced increase in TH protein expression in PC12 cells [26]. Our results consistently demonstrated that treatment with *L. platyphylla* extracts significantly attenuates the nicotine-induced alterations in TH and DAT expression in PC12 cells. These findings suggest that *L. platyphylla* extract contains pharmacological compounds that affect the dopaminergic system. In the analysis of *L. platyphylla* extract using UPLC-MS/MS, we found that *L. platyphylla* extract contains a large amount of spicatoside A and spicatoside D. Thus, these two saponin compounds may have a mitigating effect on the dopaminergic system altered by nicotine exposure. Spicatoside A was previously reported to promote neurite outgrowth in the PC12 cell line through the tyrosine kinase A receptor pathway [26]. Further study is required to understand the mechanisms underlying the mitigating effects of spicatoside A and spicateside D in *L. platyphylla* extract on the nicotine-induced stimulation of the dopaminergic system.

Behavioral sensitization, an addictive phenomenon, refers to the hypersensitivity of motivated behaviors after repeated exposure to psychostimulants such as nicotine [9]. Many studies have shown that the development of psychostimulant-induced behavioral sensitization is closely related to the alteration in dopaminergic neurotransmission in the reward system including in the dSTR and NAc [2,3,9,10]. Ginseng saponins and herbal extracts significantly attenuate nicotine-induced behavioral sensitization in rodents [2,4,8]. Ginsenoside Rb2 was considered to inhibit adenylate cyclase related to nicotine-induced dopaminergic activation [8,27]. Ginsenoside Rc in ginseng regulates the GABA_A_ receptor in the brain [4,28]. Tachikawa et al. reported that ginsenoside Rg2 decreased the secretion of catecholamines induced by nicotine from the adrenal chromaffin cells of guinea pigs [29]. In this study, pretreatment with *L. platyphylla* extract significantly and consistently attenuated the repeated nicotine-induced behavioral sensitization. Taken together, these finding suggested that *L. platyphylla* extract has a mitigating effect on nicotine-induced behavioral sensitization.

Unlike our in vitro results, the in vivo approaches in this study demonstrated that repeated nicotine administration significantly increased TH protein expression in the dSTR, but DAT protein expression in the dSTR was not altered. However, the level of DAT protein expression in the NAc was significantly decreased after repeated nicotine administration, but TH protein expression in the NAc was not altered. Previous findings demonstrate that nicotine-induced dopamine signaling and neuroadaptation are regulated by activation of nicotinic acetylcholine receptors in a region-specific manner [30,31,32]. Taken together, these findings suggest that nicotine may differentially regulate TH and DAT protein expression in the dopaminergic system in a region-specific manner, leading to behavioral sensitization. Additionally, consistent with the in vitro study, the results demonstrated that pretreatment with *L. platyphylla* extract significantly rescued the repeated nicotine-induced decrease in the level of DAT protein expression in the NAc. However, pretreatment with *L. platyphylla* extract did not mitigate the repeated nicotine-induced increase in TH protein expression in the dSTR. These findings suggest that the major saponin compounds in *L. platyphylla* extracts—spicatoside A and spicatoside D—may pharmacologically act on the dopaminergic system, mitigating the nicotine-induced behavioral sensitization through regulation of DAT protein expression in the NAc. Based on previous findings [33,34,35], it could be thought that the inconsistency between in vitro and in vivo results of this study was due to differences in the duration and frequency of nicotine exposure, doses of nicotine, metabolic rate, and other in vivo conditions. Additionally, there is a limitation of this study that the mitigating effects of *L. platyphylla* extract on nicotine-induced behavioral sensitization was investigated by only in the level of TH and DAT protein expression. It is well-known that level of mRNA expression is generally correlated to protein expression [36], and ginseng saponins were found to regulate nicotine-induced alteration in dopamine-related mRNA expression [26]. Therefore, in-depth approaches for exploring the effects of saponin compounds (spicatoside A and spicatoside D) on nicotine-induced changes in mRNA expression of the reward system with a study for the reward circuit are needed in further study.

## 5. Conclusions

In conclusion, this paper reported the activity of *L. platyphylla* extract that attenuated nicotine-induced behavioral sensitization. The nicotine-induced increase in TH protein expression was decreased and nicotine-induced decrease of DAT protein expression was increased by treatment with *L. platyphylla* extract in PC12 cells. *L. platyphylla* extract attenuated the repeated nicotine-induced locomotor hyperactivity in rats by regulating DAT protein expression in the NAc. UPLC-MS systems were used for the quantification of spicatoside A and D in *L. platyphylla* extract. These results suggest that it will be possible to develop *L. platyphylla* as a material for mitigating the effects of nicotine-induced psychomotor behaviors, and that spicatoside A and D would be good sources for quality control of *L. platyphylla*. Identifying the compounds of *L. platyphylla* that can contribute to the therapeutic effects provides important information for future drug development to treat nicotine dependence.

## Figures and Tables

**Table 1:** Optimal conditions for quadrupole time-of-flight mass spectrometry (Q-TOF/MS) analysis of *L. platypylla* extract.

Ion Source	ESI Negative Mode
Source Temp. and Dissolving Temp.	120/350 °C
Cone Gas and Dissolving Gas Flow	30/500 L/h
Capillary and Cone Volt	2.2 kV/30 V
Mass Range (*m/z*)	100 to 2000
Collision Energy Range	20 to 45 eV

**Table 2:** Optimized MS/MS parameters of standard compounds.

Compounds	MRM ^a^	Time ^b^	DP ^c^	EP ^d^	CEP ^e^	CE ^f^	CXP ^g^
Spicatoside A	869.385/737.4	150	–85	–11	–38	–40	–10
Spicatoside D	1049.423/917.2	150	–110	–8.5	–32	–54	–10

^a^ MRM, multiple reaction monitoring. ^b^ Mass scan time. ^c^ DP, declustering potential. ^d^ EP, entrance potential. ^e^ CEP, collision cell entrance potential. ^f^ CE, collision energy. ^g^ CXP, collision cell exit potential.

**Table 3:** Identification of two compounds in *L. platyphylla* by UPLC-MS/MS.

Compounds	Molecular Formula	MW	Measured Value ^a^ [M–H]	MS/MS Fragmentation ^a^
**Spicatoside A**	C_44_H_70_O_17_	871.01	869.4506	737.4 [M–H–Glc]^–^; 707.2 [M–H–Glc]^–^; 575.3 [M–H–Glc–Glc]^–^; 145.1 [M–H–Glc–Glc–aglycone]^–^
**Spicatoside D**	C_50_H_82_O_23_	1051.2	1049.5100	917.2 [M–H–Glc]^–^; 887.2 [M–H–Glc]^–^; 755.3 [M–H–Glc–Glc]^–^; 609.3 [M–H–Glc–Glc]^–^; 447.0 [M–H–Glc–Glc–Glc–Glc]^–^; 403.2 [M–H–Glc–Glc–Glc–Glc–C_2_H_5_O–]^–^

^a^ Mass accuracy < 5 ppm.

**Table 4:** Linear regression data and contents of the validated method for the investigated two compounds in *L. platyphylla* ^a^.

Compounds	Rt ^b^ (min)	Calibration Curve ^c^	*R* ^2 d^	Line Arrangement (ug/mL)	LOD ^e^ (ppm)	LOQ ^f^ (ppm)	Amount (mg/g)
**Spicatoside A**	9.53	*y =* 14.4*x* + 9670	0.9948	0.625–5.000	1.382	4.188	0.148
**Spicatoside D**	3.97	*y =* 5.08*x* + 0.00266	0.9957	0.625–5.000	1.248	3.783	0.272

^a^ Mean value of samples (*n* = 3). ^b^ Rt, retention time. ^c^ *y*, logarithmic value of peak area; *x*, logarithmic value of amount injected. ^d^ R^2^, linearity. ^e^ LOD, limit of detection. ^f^ LOQ, limit of quantitation.

**Table 5:** Changes in locomotor activity following the final administration of saline or nicotine after pretreatment of vehicle or *L. platyphylla* extract.

Groups	Locomotor Activity (cm)		
Vehicle + Repeated saline	3042.94	±	389.34
200 mg/kg *L. platyphylla* extract + Repeated saline	2432.47	±	535.15
400 mg/kg *L. platyphylla* extract + Repeated saline	2153.31	±	293.55
Vehicle + Repeated nicotine	6313.58	±	1037.62 **
200 mg/kg *L. platyphylla* extract + Repeated nicotine	4112.00	±	767.80
400 mg/kg *L. platyphylla* extract + Repeated nicotine	2670.44	±	215.12 ^##^

One-way ANOVA, followed by Tukey’s post hoc test was used for statistical analysis. ** *p* < 0.01 vs. vehicle + repeated saline group. ^##^ *p* < 0.01 vs. vehicle + repeated nicotine group.
